# The economic and psychological impacts of covid-19: a exploratory study of the intersection of traditional and new vulnerable groups in South Korea

**DOI:** 10.1186/s12889-024-20424-w

**Published:** 2024-11-07

**Authors:** Yoonkyoung Lee, Taejin Lee

**Affiliations:** 1 Department of Public Health Sciences, Graduate School of Public Health, Seoul National University, Seoul, Republic of Korea; 2 Institute of Health and Environment, Seoul National University, Seoul, Republic of Korea

**Keywords:** COVID-19, Vulnerable populations, Economic impact, Psychological effects, Korean context, Survey data, Income instability, Job security, Stress levels, Policy implications

## Abstract

**Background:**

The global impact of the COVID-19 pandemic has been extensive, disproportionately affecting various vulnerable populations. In addition to traditional vulnerable groups (TVGs) such as the elderly, women, people with lower education, disabled individuals, and low-income groups, new vulnerable groups (NVGs) have emerged, including essential workers, face-to-face workers, temporary workers, and the self-employed. This study focuses on exploring the economic and psychological effects of the pandemic on both traditional and newly recognized vulnerable groups within Korea.

**Methods:**

The study employed multiple logistic regression using micro-level social survey data from Statistics Korea to calculate the odds ratio relating to two categories of vulnerable groups and their economic and mental health impacts. Additionally, through interaction term analysis, we tried to uncover the complex interrelationship between these non-vulnerable groups and target vulnerable groups.

**Results:**

Both the newly identified and traditional vulnerable groups showed higher likelihood of facing economic and mental health challenges compared to non-vulnerable groups. The likelihood of encountering a decrease in income and work-related stress was 2.17 times higher (95% CI = 1.90–2.47) for NVGs, while those belonging to TVGs had a 1.75-fold increase (95% CI = 1.47–2.08). Moreover, female self-employed workers faced higher overall stress at 1.36 times greater risk (95% CI = 1.08–1.71), whereas elderly individuals who are self-employed exhibited lower odds of experiencing such stress at the intersection between TVGs and NVGs.

**Conclusion:**

This research offers in-depth understanding of the diverse impacts of COVID-19 on at-risk groups. Furthermore, examinations that take into account interactions between NVGs and TVGs uncovered subtle effects within subgroup dynamics, suggesting that intersecting factors play a role in different levels of vulnerability. This provides valuable evidence for informing policy decisions and allocating resources.

**Supplementary Information:**

The online version contains supplementary material available at 10.1186/s12889-024-20424-w.

## Background

The coronavirus disease 2019 (COVID-19) pandemic has profoundly impacted lives and livelihoods worldwide. Recent data indicated over 775 million confirmed cases globally, with more than seven million recorded deaths [1]. Additionally, from 2019 to 2020, global unemployment surged from 191.9 million to 235.2 million, marking the largest yearly rise since 1991 [2]. Additionally, the World Health Organization reports a 25% increase in the prevalence of anxiety and depression worldwide since the pandemic’s onset [3].

Amidst this crisis, it is crucial to acknowledge that these burdens are not equally distributed [4–7], falling disproportionately on populations that are already vulnerable, exacerbating existing disparities.

Among those most affected are traditionally marginalized and disadvantaged groups including the elderly, women and the disabilities [8]. For instance, older adults face heightened risks as a result of age-related health concerns [9, 10], while women endure disproportionate impacts as a result of caregiving responsibilities and systemic gender inequalities [11, 12]. Individuals with disabilities encounter exacerbated challenges accessing healthcare and employment opportunities, even during normal circumstances [13, 14]. Furthermore, those from low-income backgrounds are further pushed into economic instability by the pandemic’s disruptions in employment and financial support systems [15, 16].

However, beyond traditional vulnerabilities, the pandemic has unveiled new groups of vulnerable people, highlighting segments of society that were previously overlooked. These new vulnerable groups(hereafter, NVGs) include essential workers, who maintain critical services despite facing daily risks to their health and safety. This group comprises not only frontline healthcare workers but also grocery store staff and other critical service providers [17, 18]. Face-to-face workers, who must interact directly with others, are constantly exposed to the threat of the virus in their workplaces [19, 20]. Temporary workers face increased uncertainty, as economic downturns and fluctuations in demand make their job prospects unstable [21–24]. Additionally, self-employed individuals, including small business owners and freelancers, have been particularly hard-hit by lockdowns and containment measures, facing not only financial challenges but also long-term mental health consequences [25–29]. It is important to recognize that these two groups—traditional vulnerable groups(hereafter, TVGs) and the newly vulnerable groups—are not discrete entities, but are rather intricately interconnected. Therefore, they are intertwined in a complex web of interdependence, where each group profoundly influences the others. This underscores the nuanced dynamics of vulnerability in contemporary society, requiring a comprehensive understanding of how they interact and impact each other.

Despite the urgent need to address these multifaceted vulnerabilities, existing research has often focused narrowly on individual groups, overlooking the complex interactions and cumulative impacts spanning various vulnerable populations. This oversight not only undermines individuals’ ability to effectively respond to the current crisis but also hampers preparedness for future infectious disease outbreaks.

Recognizing the need for a more comprehensive approach, this study aimed to fill this gap by exploring the impact of COVID-19 on both TVGs and NVGs, with particular emphasis on the economic and psychological ramifications. Delving into the intricate interplay between these two spheres of vulnerability, this study seeks to shed light on their interconnectedness and implications. By examining the multifaceted effects of the pandemic on individuals from diverse socioeconomic backgrounds, including essential workers, frontline personnel, temporary workers, and self-employed individuals, alongside marginalized populations such as females, low-income households, and the elderly, this study endeavors to provide a holistic understanding of the complex dynamics at play.

## Methods

### Data

The researchers utilized micro-level social survey data from Statistics Korea employing the Micro Data Integrated Service (MDIS) from Statistic Korea. This was an individual-level interview survey of approximately 32,000 households nationwide [30]. The survey was stratified into 27 districts reflecting regional characteristics, and data were collected utilizing two-stage cluster sampling. In odd-numbered years, the survey covered welfare, social participation, leisure, income, consumption, and labor, whereas, in even-numbered years, it covered health, education and training, crime and safety, and family as well as living environments. In response to user requests, the social survey included questions related to changes in life as a result of only COVID-19 in the 2021 and 2022 surveys when the number of infections in South Korea surged.

In this study, we utilized social survey data from 2021 to 2022 to examine changes in the lives of NVGs as a result of COVID-19. The data were anonymized prior to being accessed by the research team. This study was exempt from the approval by the Institutional Review Board (IRB) at Seoul National University (IRB No. E2110/002–010).

### Variables

#### Dependent variable

This study aims to explore the financial and psychological impacts of COVID-19. Economic effects can be broadly categorized into income loss and job instability. To assess income loss, we utilized a 5-point Likert scale to ask participants about changes in their household income compared to a year ago (“Have you experienced a reduction in earnings compared to 2020?“). If the response indicated as “slightly decreased” or “significantly decreased,” the participants were then asked whether this decline was primarily linked to COVID-19 (“Is this decline mainly related to COVID-19?“), enabling for a “Yes” or “No” response. In this study, experiences of income loss as a result of COVID-19 were categorized based on this response as a binary variable. Similarly, job instability was evaluated by asking participants if they felt anxious about potential job loss or needing new employment in the near future utilizing a 4-point scale (“Do you often experience anxiety about possible job loss or having to find new employment soon?“). In addition, the link to COVID-19-related unease among those who answered “feel very much” or “feel somewhat,” was explored. Job instability was coded as a binary variable based on this response.

In terms of the psychological effects, we looked specifically at stress in different contexts—household, workplace, and overall daily life—to investigate the influence of COVID-19-related stress. While the survey initially included stress related to school life, it was later excluded from the analysis due to the age-related characteristics of vulnerable groups. To accomplish this, a survey with a 5-point scale was utilized to evaluate participants’ level of stress during their daily lives (“How much stress have you felt in your daily life?“). Subsequently, individuals who indicated either “experienced a lot” or “experienced somewhat” on the scale were targeted and further examined whether their primary source of stress was associated with COVID-19 (“Was the main reason for your stress related to COVID-19?“). Based on the responses to this question, the occurrence of COVID-19-induced stress was classified as a binary variable within this study.

#### Independent variable

Both the TVGs known to be susceptible to disasters and the NVGs that emerged during infectious disease outbreaks were considered. There are five traditional vulnerable groups: the elderly, women, individuals with disabilities, low-income individuals, and those with low levels of education. NVGs were categorized based on Professor Robert Reich’s classification [31] and included essential workers, face-to-face workers, temporary workers, and self-employed workers. NVG and TVG are defined as follows.

#### Newly vulnerable group (NVG)

##### Essential worker

The delineation of essential workers exhibits variability across nations and disaster contexts and lacks a uniform consensus. Following the onset of the COVID-19 pandemic, several major countries such as the United States, Italy, and the United Kingdom enacted stringent measures to curtail specific industries, aiming to mitigate disease transmission while exempting sectors deemed essential for meeting fundamental human needs. Sanchez et al. [32] identified essential sectors based on these governmental directives, encompassing domains like healthcare, food provision, utilities (electricity, gas, water), and transportation [32]. In this study, the classification of essential labor occupations was drawn from the North American Industry Classification System (NAICS) as delineated by Sanchez et al. [32] and subsequently mapped onto the Korean Standard Industrial Classification (KSIC) [33]. Although the definition of essential labor may exhibit situational and country-specific nuances, the framework proposed by Sanchez et al. [32] represents a conservative approach, targeting occupational categories deemed essential across Italy and three U.S. states (Delaware, Minnesota, and Oklahoma).

##### Face-to-face worker

Dingel and Neiman [34] and Mongey et al. (2020) assessed the correlation between telework availability and infectious diseases by computing a telework availability index for each occupational category based on job functions and characteristics sourced from the U.S. O*Net survey aligned with the U.S. Standard Occupational Classification (SOC) [34, 35]. The feasibility of telecommuting is gauged by factors such as remote work compatibility and equipment provision [33]. In the Korean context, Oh et al. (2020) evaluated employment vulnerability to COVID-19 by cross-referencing the SOC-derived telecommuting index proposed by Dingel and Neiman [34] with subclassifications from the Korean Standard Classification of Occupations (KSCO) [33, 34]. In this study, face-to-face workers were distinguished by utilizing the criteria outlined by Dingle and Neiman [34] and matched them to the Korean context, as proposed by Oh et al. (2020).

##### Precarious labor

With regard to precarious workers, temporary work and self-employment were utilized as variables. The distinction between temporary workers and self-employed individuals was based on respondents’ answers to the survey questions. With regard to temporary workers, respondents who answered “temporary worker” or “day laborer” to the question regarding employment status were coded as 1, while all other responses were coded as 0. Self-employed individuals were identified by responses indicating “self-employed with employees” or “self-employed without employees” to questions in relation to employment status, which were coded as 1, while other responses were coded as 0.

#### Traditional vulnerable group (TVG)

TVGs were selected based on specific criteria. Individuals aged 65 or older were considered elderly, while those with less than a high school diploma were categorized as having lower levels of education. For individuals with disabilities, classification was contingent on the possession of a disability welfare card. For the low-income group, the average per capita income was computed based on household size and income. Additionally, the analysis categorized low-income households as those earning less than 50% of the median income of a family of four.

#### Control variable

We defined sex as a binary variable: female or male. We defined age as a continuous variable. Educational attainment was a categorical variable ranging from 1 to 7. Household income was coded in units of 1 million won, ranging from 1 (less than 1 million won) to 9 (800 million won or more), standardized to household income based on a household size of four.

### Statistical analysis

This study utilized multiple logistic regression analyses to examine the economic and psychological repercussions of COVID-19 across diverse vulnerable demographics. The comparative analysis of five types of TVGs and four categorized NVGs variables respectively poses a challenge due to the variability in their reference groups. Therefore, we created a single variable utilizing a common reference group (individuals not belonging to any of the nine identified vulnerable groups) to assess how each group was affected. This reference group, or control group, consists of individuals who do not fall into any of the five traditional vulnerable groups (elderly, female, less educated, disabled, and low income) nor into any of the four new vulnerable groups (essential workers, face-to-face workers, temporary workers, and self-employed workers).

Additionally, this study examined the intersectionality within these vulnerable groups, considering factors such as age, income, education, and sexual orientation to understand how multiple dimensions of vulnerability intersect and compound the challenges faced by individuals. All analyses were conducted utilizing the Stata 17. Two-sided p-values of 0.05 were considered statistically significant. However, given the exploratory nature of this study, a *p* < 0.1 threshold was also used to identify potentially significant trends.

## Results

### Baseline characteristics

Table 1 presents summary statistics regarding sociodemographic characteristics of the study participants. This study involved 36,423 participants in 2021 and 39,721 participants in 2022. Among the study participants in 2021 and 2022, males were 17,672 (48.52%) and 19,274 (48.52%) respectively. In terms of work status, 14,488 (39.78%) and 14,047 (35.36%) were wage workers while 16,052 (55.93%) and 19,696 (49.59%) were unemployed. Approximately 13,184 (36.20%) and 13,146 (36.73%) had attained college degree or higher education. Furthermore, 5,342 (14.67%) and 6,336 (17.70%) belonged to the richest househods based on the household income.

### Multiple logistic regression for COVID-19 impacts of vulnerable group

Table 2 exhibits the economic and psychological impacts of COVID-19 on NVGs, which are newly defined by employment characteristics, and the existing TVGs.

The likelihood of facing a decrease in income or increased stress at work owing to COVID-19 was higher for NVGs than for TVGs. Specifically, the odds of experiencing a reduction in income as a result of COVID-19 were 2.17 (95% CI = 1.90–2.47) times higher, and the odds of experiencing work-related and overall stress were 2.96 (95% CI = 2.61–3.70) times and 1.15 (95% CI = 1.03–1.29) times higher respectively for NVGs. Conversely, job instability as a result of COVID-19 was more prevalent among TVGs (OR = 1.77, 95% CI = 1.50–2.10) than NVGs (OR = 1.52, 95% CI = 1.35–1.70), while differences in household stress levels were not significant when compared with the reference group for either category.

**Table 1:** Sociodemographic characteristics of the study participants

Variable		*N*		%	
		2021	2022	2021	2022
All		36,423	39,721	100.00	100.00
Age					
	13–19	2,525	2,357	6.93	6.59
	20–29	4,270	3,811	11.72	10.65
	30–39	4,666	4,423	12.81	12.36
	40–49	6,385	6,059	17.53	16.93
	50–59	6,558	6,502	18.01	18.17
	60 and over	12,019	12,640	33.00	35.32
Elderly Status					
	Below 65	27,890	30,601	76.57	77.04
	65 and over	8,533	9,120	23.43	22.96
Sex					
	Male	17,672	19,274	48.52	48.52
	Female	18,751	20,447	51.48	51.48
Temporary worker					
	Yes	4,004	3,612	10.99	9.09
	No	32,419	36,109	89.01	90.91
Work status					
	Wage worker	14,488	14,047	39.78 (71.12)	35.36 (70.15)
	Employer	1,280	12,282	3.51 (6.28)	3.23 (6.40)
	Self employed	3,520	3,594	9.66 (17.28)	9.05 (17.95)
	Unpaid family worker	1,083	1,102	2.97 (5.32)	2.77 (5.50)
	Unemployed	16,052	19,696	55.93	49.59
Disability					
	Yes	2,227	2,250	6.11	6.29
	No	34,196	33,542	93.89	93.71
Educational level					
	Under elementary	6,055	6,102	16.62	17.05
	Middle school graduates	4,429	4,250	12.16	11.87
	High school graduates	12,755	12,294	35.02	34.35
	College degree or higher	13,184	13,146	36.20	36.73
Household income					
	Under 1 million won(KRW)	5,135	4,831	14.10	13.50
	1 million – 2 million won	5,995	5,312	16.46	14.84
	2 million – 3 million won	6,776	6,056	18.60	16.92
	3 million – 4 million won	5,632	5,480	15.46	15.31
	4 million – 5 million won	4,413	4,325	12.12	12.08
	5 million – 6 million won	3,130	3,452	8.59	9.64
	6 million won and over	5,342	6,336	14.67	17.70

**Table 2:** Multiple logistic regression analysis of the impact of COVID-19 on vulnerable groups

	Economic impacts		Psychological impacts (Stress)		
	Job Instability	Income loss	Household	Work	Overall
	OR(95% CI)	OR(95% CI)	OR(95% CI)	OR(95% CI)	OR(95% CI)
Non-vulnerable group	1.00	1.00	1.00	1.00	1.00
New vulnerable group	1.52*** (1.35–1.70)	2.17*** (1.90–2.47)	0.94 (0.81–1.09)	2.96*** (2.61–3.70)	1.15* (1.03–1.29)
Traditional vulnerable group	1.77*** (1.50–2.10)	1.75*** (1.47–2.08)	1.11 (0.95–1.30)	1.26** (1.07–1.49)	1.01 (0.88–1.14)

### Multiple logistic regression of COVID-19 impacts for a specific vulnerable group

Table 3 presents more detailed results regarding the impact of COVID-19 on the two vulnerable groups. According to the table, self-employed individuals exhibited the highest odds of experiencing job instability (OR = 2.18, 95% CI = 1.87–2.53), followed by the elderly (OR = 1.89, 95% CI = 1.48–2.42) and those with low income (OR = 1.78, 95% CI = 1.27–2.50). Self-employed individuals also had the highest odds of experiencing workplace stress (OR = 3.96, 95% CI = 3.40–4.61) and overall stress (OR = 1.54, 95% CI = 1.33–1.77) as a result of COVID-19, indicating their significant vulnerability to the pandemic. Conversely, for temporary workers, the odds of experiencing job instability (OR = 1.07, 95% CI = 0.89–1.29) were not significant, whereas the odds of experiencing income loss due to COVID-19 were significantly higher (OR = 3.10, 95% CI = 2.56–3.75). Regarding mental health, temporary workers ranked second highest among the nine vulnerable groups in terms of workplace stress (OR = 2.86, 95% CI = 2.35–3.48) as a result of COVID-19, and their overall stress odds were notably high (OR = 1.22, 95% CI = 1.00-1.47), trailing behind only the self-employed (OR = 1.54, 95% CI = 1.33–1.77) and low-income groups (OR = 1.43, 95% CI = 1.17–1.75).

For the low-income group, it was observed that they had higher odds of experiencing negative outcomes in both the two economic impact variables and the three mental health variables examined in this study.

**Table 3:** Multiple logistic regression analysis of the impact of COVID-19 on vulnerable groups (detailed)

		Economic impacts		Psychological impacts (Stress)		
		Job Instability	Income loss	Household	Work	Overall
		OR(95% CI)	OR(95% CI)	OR(95% CI)	OR(95% CI)	OR(95% CI)
Non-vulnerable group		1.00	1.00	1.00	1.00	1.00
New Vulnerable group	Essential worker	1.51*** (1.25–1.82)	0.73* (0.57–0.94)	0.85 (0.62–1.16)	2.47** (1.99–3.08)	0.91 (0.72–1.15)
	Face to face Worker	1.26** (1.09–1.44)	1.43*** (1.22–1.68)	0.75** (0.61–0.92)	2.34*** (1.99–2.74)	0.87† (0.74-1.00)
	Temporary Worker	1.07 (0.89–1.29)	3.10*** (2.56–3.75)	1.07 (0.83–1.38)	2.86*** (2.35–3.48)	1.22* (1.00-1.47)
	Self-employed Worker	2.18*** (1.87–2.53)	3.92*** (3.37–4.55)	1.07 (0.89–1.30)	3.96*** (3.40–4.61)	1.54*** (1.33–1.77)
Traditional Vulnerable group	Elderly	1.89*** (1.48–2.42)	1.45** (1.14–1.83)	0.86 (0.69–1.08)	0.87 (0.69–1.10)	0.78* (0.64–0.94)
	Female	1.71*** (1.33–2.19)	2.27*** (1.76–2.91)	1.20† (1.00-1.45)	1.75*** (1.41–2.16)	1.09 (0.93–1.28)
	Less educated	1.41† (1.00–2.00	4.00*** (2.84–5.62)	1.14 (0.89–1.47)	2.65*** (1.82–3.87)	1.18 (0.94–1.48)
	Disabled	1.63*** (1.27–2.08)	1.91*** (1.50–2.43)	1.01 (0.80–1.26)	1.41** (1.10–1.80)	1.04 (0.86–1.26)
	Low income	1.78** (1.27–2.50)	3.52*** (2.56–4.85)	1.69*** (1.34–2.14)	1.64** (1.23–2.20)	1.43*** (1.17–1.75)

Abbreviations OR = odds ratio; CI = confidence interval

* Adjusted for age, sex, income, and educational level

†<0.1 *<0.05 **<0.01 ***<0.001

For older individuals, the odds of experiencing negative economic effects, such as job instability (OR = 1.89, 95% CI = 1.48–2.42) and income loss (OR = 1.45, 95% CI = 1.14–1.83), as a result of COVID-19 were significantly higher. However, the odds of experiencing overall stress (OR = 0.78, 95% CI = 0.64–0.94) were lower. Women were more susceptible to the negative economic and psychological effects of COVID-19, especially household stress, with significantly higher odds (OR = 1.20, 95% CI = 1.00–1.45) compared to non-vulnerable groups. Finally, for those with lower educational levels or disabilities, the odds of experiencing negative economic impacts were high, especially for income loss. In addition, they had high odds of experiencing workplace stress.

### Comparative analysis of COVID-19 impact across TVGs and NVGs intersections

In general, TVGs are associated with individual socioeconomic factors that also impact employment. Consequently, a higher proportion of TVGs such as older individuals and women are often found in occupations that are relatively unstable or susceptible to infection [36]. Therefore, in this section, a subgroup analysis was conducted by incorporating an interaction term between NVGs and TVGs to delve deeper into the intricacies within these subgroup dynamics.

As shown in Table 4, the analysis revealed that, among the elderly, working in temporary positions was associated with a decrease in the odds of experiencing job instability (OR = 0.59, 95% CI = 0.34–1.02) or income reduction (OR = 0.32, 95% CI = 0.18–0.56), as well as a decrease in the odds of experiencing overalll stress (OR = 0.77, 95% CI = 0.59-1.00). Furthermore, elderly self-employed individuals exhibited significantly lower odds of experiencing workplace stress (OR = 0.58, 95% CI = 0.35–0.95) or overall stress (OR = 0.73, 95% CI = 0.59–0.90), and although not significant, there was a tendency towards lower odds of experiencing household stress (0.87, 95% CI = 0.68–1.13).

For essential female workers and face-to-face workers, there was a significant decrease in the odds of experiencing job instability, with ORs of 0.70 and 0.75, respectively. Conversely, self-employed women had significantly higher odds of experiencing work-related stress (OR = 1.26, 95% CI = 1.02–1.57) and overall stress (OR = 1.36, 95% CI = 1.08–1.71) as a result of COVID-19. This finding contrasts with the results observed among self-employed elderly individuals and those with lower levels of education, where a different pattern was evident.

In addition, the interaction between disability and NVGs revealed a significant decrease in psychological impact, whereas there was a notable increase in economic shocks. Particularly, essential workers with disabilities exhibited a 4.77-fold increase in job instability (95% CI: 1.00-22.78), which was significantly higher compared to their non-disabled counterparts.

Among the essential workers, those with lower levels of education or income were found to be 2.95–3.73 times more likely to experience workplace stress as a result of COVID-19.

## Discussion

This study investigated the economic and psychological impact of COVID-19 on the NVG and TVG in South Korea. A multiple logistic regression analysis was utilized to examine the odds of experiencing job instability, income loss, and stress as a result of COVID-19. To the best of our knowledge, this study is a pioneering investigation into the intricate interplay between TVGs and NVGs in the context of the COVID-19 pandemic, shedding light on their interconnectedness and implications.

**Table 4:** Intersecting vulnerability profiles: Traditional and New Vulnerability Group Dynamics in the Context of COVID-19

**a. Economic Impacts**												
	**Job instability**				**Income loss**							
	**Essential Worker**	**F2F worker**	**Temporary worker**	**Self-employed worker**	**Essential worker**	**F2F worker**	**Temporary worker**	**Self-employed worker**				
	**OR (95% CI)**	**OR (95% CI)**	**OR (95% CI)**	**OR (95% CI)**	**OR (95% CI)**	**OR (95% CI)**	**OR (95% CI)**	**OR (95% CI)**				
Elderly	1.39 (0.28–6.99)	0.63 (0.35–1.13)	0.59+ (0.34–1.02)	0.82 (0.48–1.43)	2.04 (0.60–6.96)	0.64 (0.35–1.16)	0.32*** (0.18–0.56)	0.35*** (0.20–0.59)				
Female	0.70* (0.49–0.99)	0.75† (0.55–1.01)	1.07 (0.80–1.42)	0.99 (0.72–1.36)	1.15 (0.75–1.75)	0.95 (0.69–1.32)	0.79 (0.59–1.07)	1.26 (0.94–1.70)				
Less educated	-	0.77 (0.35–1.71)	0.94 (0.43–2.05)	1.07 (0.49–2.32)	-	0.63 (0.27–1.44)	0.32** (0.14–0.72)	0.35* (0.15–0.78)				
Disabled	4.77† (1.00-22.78)	1.10 (0.55–2.21)	0.98 (0.51–1.88)	1.28 (0.67–2.46)	2.18 (0.67–7.02)	1.08 (0.48–2.44)	0.72 (0.33–1.56)	0.84 (0.40–1.74)				
Low income	0.15 (0.15–1.48)	0.30 (0.06–1.63)	0.24† (0.05–1.10)	0.32 (0.07–1.46)	0.20 (0.02–2.34)	0.87 (0.25–3.10)	0.23** (0.08–0.64)	0.19** (0.07–0.52)				
**b. Psychological Impacts**												
	**Household**				**Workplace**				**Overall**			
	**Essential worker**	**F2F worker**	**Temporary worker**	**Self-employed worker**	**Essential worker**	**F2F worker**	**Temporary worker**	**Self-employed worker**	**Essential worker**	**F2F worker**	**Temporary worker**	**Self-employed worker**
	**OR (95% CI)**	**OR (95% CI)**	**OR (95% CI)**	**OR (95% CI)**	**OR (95% CI)**	**OR (95% CI)**	**OR (95% CI)**	**OR (95% CI)**	**OR (95% CI)**	**OR (95% CI)**	**OR (95% CI)**	**OR (95% CI)**
Elderly	1.70 (0.50–5.77)	1.15 (0.84–1.57)	0.83 (0.62–1.12)	0.87 (0.68–1.13)	1.58 (0.60–4.21)	1.00 (0.60–1.67)	0.71 (0.42–1.19)	0.58* (0.35–0.95)	1.31 (0.45–3.80)	1.26 (0.97–1.64)	0.77* (0.59-1.00)	0.73** (0.59–0.90)
Female	0.77 (0.51–1.17)	0.98 (0.72–1.33)	0.86 (0.63–1.18)	1.11 (0.83–1.50)	1.20 (0.91–1.59)	0.99 (0.79–1.24)	0.91 (0.72–1.17)	1.26* (1.02–1.57)	1.22 (0.89–1.66)	1.07 (0.84–1.36)	1.11 (0.86–1.43)	1.36** (1.08–1.71)
Less educated	1.67 (0.20-14.08)	1.23 (0.94–1.62)	0.92 (0.70–1.21)	0.95 (0.74–1.22)	3.73† (0.87–16.05)	0.95 (0.50–1.78)	0.66 (0.35–1.26)	0.57† (0.31–1.07)	4.40* (1.06–18.26)	1.43** (1.14–1.80)	0.75* (0.58–0.97)	0.83† (0.67–1.03)
Disabled	0.98 (0.34–2.80)	0.49* (0.24–0.99)	0.59† (0.32–1.07)	0.58* (0.36–0.96)	0.42† (0.16–1.08)	0.47* (0.24–0.92)	0.50† (0.25–1.01)	0.55* (0.30-1.00)	0.54 (0.21–1.39)	0.52* (0.30–0.89)	0.51* (0.30–0.86)	0.77 (0.54–1.09)
Low income	0.73 (0.14–3.88)	0.92 (0.37–2.31)	0.46 (0.18–1.16)	0.66 (0.28–1.59)	2.95† (0.92–9.46)	0.99 (0.39–2.49)	1.04 (0.43–2.51)	0.75 (0.32–1.77)	1.94 (0.59–6.33)	0.77 (0.32–1.86)	0.68 (0.30–1.55)	0.81 (0.37–1.77)

Abbreviations OR = odds ratio; CI = confidence interval

* Adjusted for age, sex, income, and educational level

†<0.1 *<0.05 **<0.01 ***<0.001

Both the NVG and TVG experienced significantly higher odds of economic shocks, such as job instability or income loss as a result of COVID-19, as opposed to non-vulnerable groups. While job instability was more pronounced in TVGs, NVGs were faced with higher income loss. Despite no significant increase in household stress or general stress levels, there was a notable surge in workplace stress, particularly among NVGs.

A detailed examination of COVID-19’s impact on each vulnerable group highlighted significant economic and psychological challenges faced by self-employed workers. This finding can be elucidated through the job-demand model, which posits that the balance between job demands and job control plays a crucial role in determining stress levels and, ultimately, health outcomes [37]. When this model was applied to the context of COVID-19, self-employed individuals typically preferred higher job control as opposed to wage workers. However, during the COVID-19 pandemic, government measures, such as restrictions on business activities or enforced temporary closures, sharply reduced their job control. This sudden loss of control, coupled with heightened job demands (such as maintaining business operations in uncertain conditions), led to increased stress [25]. The loss of job control is particularly impactful on health because it removes a critical coping mechanism that helps individuals manage stress [38]. Our findings align with previous research documenting the negative impact of COVID-19 on self-employed workers’ health. Studies by Blundell et al. [39] and Adams-Prassl et al. [29] found that self-employed individuals across the UK and Europe faced heightened psychological stress and job insecurity due to financial instability and loss of job control, reinforcing the importance of job control as a protective factor [29, 39].

Essential workers were the only group encompassing both the NVG and TVG to exhibit lower odds of experiencing income loss as a result of COVID-19, which can be attributed to the essential nature of their occupations, regardless of the circumstances. However, they experienced heightened workplace stress as a result of the pandemic, particularly essential workers with lower educational levels, indicating significantly higher odds of experiencing stress. Temporary workers were the only group that exhibited an insignificant effect of job instability. This may be a result of their inherently higher levels of job instability.

In addition, the researchers examined whether there were differences within each NVG, utilizing the interaction term with the TVG. The results exhibited that self-employed women are most likely to experience general stress and workplace stress. The heightened deterioration of mental health among self-employed women aligns with existing research on the health of self-employed individuals [25–27]. This may be attributed to increased caregiving responsibilities as a result of government containment measures, such as school closures or the transition to remote learning, which disproportionately impacted women during pandemic [25]. Several studies have documented that women, particularly those who are self-employed or work in precarious conditions, experienced greater stress due to the dual burden of work and caregiving during COVID-19 [40, 41]. This observation is further supported by our findings, which exhibited a significant higher odds of experiencing household stress among women with COVID-19. In contrast, self-employed elderly workers exhibited lower odds of experiencing stress as a result of COVID-19. Traditionally, older adults have been considered vulnerable to disasters because of their physical fragility. However, in prolonged infectious disease scenarios, the elderly may exhibit mental health resilience based on a wealth of life experiences. Drawing from a myriad of experiences spanning various challenges and adversities, the elderly demonstrate a level of psychological robustness that contrasts with that of younger age groups [42].

Furthermore, there were varied impacts within the essential worker category depending on their interaction with different factors. Unlike other groups with relatively similar characteristics, essential workers encompass a wide range of occupations, from those requiring high levels of education and societal respect (e.g., healthcare professionals) to those with contrasting attributes. The diverse occupational makeup within the essential worker category may elucidate these differences. Consequently, despite belonging to the same essential worker group, different levels of societal treatment and respect can lead to varying outcomes.

There are several limitations to this analysis. First, due to the nature of cross sectional study, we can only speak to observed associations which means cannot demonstrate cause-and-effect link. Second, the reliance on a single-item question to assess the psychological impact of COVID-19 may introduce limitations in robustness. Third, apart from the economic and psychological impacts on individuals, COVID-19 can also have long-term effects on physical health, such as sequelae. However, these aspects were not considered in this study. Lastly, the economic and psychological impacts of COVID-19 on individuals may vary depending on various environmental factors such as individual asset levels and dual-income status. This study did not control for these factors due to limitations in the data.

Despite these limitations, the findings contribute to the existing literature by comprehensively examining the economic and mental impact of COVID-19 on vulnerable groups. Health and economic damages resulting from pandemics do not cease abruptly with the termination of an outbreak; rather, they persistently impact individuals’ lives and may reverberate over time. Therefore, the long-term ramifications of these effects are significant, and meticulous management of various vulnerable demographics remains imperative. Moreover, analyzing the comprehensive, collective, and holistic impact of variously constituted vulnerable populations can serve as crucial empirical evidence for strategically allocating resources to those most in need within a given budget during potential future pandemics.

## Conclusion

This study sheds light on the complex interplay between TVGs and NVGs in the midst of the COVID-19 pandemic in Korea. The results show that both NVGs and TVGs were significantly more likely to experience economic shocks, such as job instability and income loss, than non-vulnerable groups. Moreover, even within the same vulnerable population, additional vulnerabilities may be compounded, leading to differential impacts. Given the heterogeneity within vulnerable groups, such as the elderly, women and the self-employed, one-size-fits-all policies based on a single criterion may not be effective. Targeted measures are therefore needed, including flexible financial support and tailored mental health services for the self-employed and precarious workers, as well as workplace flexibility for women with caring responsibilities. Strengthening social protection for the elderly and disabled is also crucial to reduce their vulnerability in future crises. Priority should be given to policies that address the compounding nature of vulnerabilities in the event of future pandemics.

## Electronic supplementary material

Below is the link to the electronic supplementary material.

Supplementary Material 1

## Data Availability

The datasets used and analyzed during the current study are available from the corresponding author upon reasonable request.

## Abbreviations

TVG: Traditional vulnerable groups
NVG: Newly recognized vulnerable groups
MDIS: Micro Data Integrated Service
