# Supplementary material for: The economic and psychological impacts of covid-19: a exploratory study of the intersection of traditional and new vulnerable groups in South Korea
Source: BMC Public Health. 2024 Nov 7;24:3085. doi: 10.1186/s12889-024-20424-w (PMC11545496; doi:10.1186/s12889-024-20424-w)
Supplement: Supplementary file 1 — Supplementary Material 1 [file 12889_2024_20424_MOESM1_ESM.docx]

**Supplementary. Correlation between vulnerable groups**

Table S1a. Correlation between vulnerable groups (2021)

| 2021 | Temporary worker | Self-employed | Essential worker | Face to face  worker | The elderly | Female | The disabled | The less educated | Low income group |
| --- | --- | --- | --- | --- | --- | --- | --- | --- | --- |
| Temporary worker | 1.00 |  |  |  |  |  |  |  |  |
| Self-employed | -0.27 | 1.00 |  |  |  |  |  |  |  |
| Essential worker | 0.03 | -0.13 | 1.00 |  |  |  |  |  |  |
| Face to Face worker | 0.23 | 0.11 | -0.07 | 1.00 |  |  |  |  |  |
| The elderly | 0.13 | 0.17 | 0.03 | 0.29 | 1.00 |  |  |  |  |
| Female | 0.10 | -0.16 | 0.10 | -0.08 | 0.02 | 1.00 |  |  |  |
| The disabled | 0.03 | 0.07 | 0.00 | 0.09 | 0.17 | -0.06 | 1.00 |  |  |
| The less educated | 0.15 | 0.12 | -0.00 | 0.36 | 0.56 | 0.12 | 0.14 | 1.00 |  |
| Low income group | 0.09 | 0.03 | 0.01 | 0.09 | 0.16 | 0.02 | 0.05 | 0.15 | 1.00 |

Table S1b. Correlation between vulnerable groups (2022)

| 2022 | Temporary worker | Self-employed | Essential worker | Face to face  worker | The elderly | Female | The disabled | The less educated | Low income group |
| --- | --- | --- | --- | --- | --- | --- | --- | --- | --- |
| Temporary worker | 1.00 |  |  |  |  |  |  |  |  |
| Self-employed | -0.27 | 1.00 |  |  |  |  |  |  |  |
| Essential worker | 0.03 | -0.13 | 1.00 |  |  |  |  |  |  |
| Face to Face worker | 0.23 | 0.11 | -0.07 | 1.00 |  |  |  |  |  |
| The elderly | 0.13 | 0.17 | 0.03 | 0.29 | 1.00 |  |  |  |  |
| Female | 0.10 | -0.16 | 0.10 | -0.08 | 0.02 | 1.00 |  |  |  |
| The disabled | 0.03 | 0.07 | 0.00 | 0.09 | 0.17 | -0.06 | 1.00 |  |  |
| The less educated | 0.15 | 0.12 | -0.00 | 0.36 | 0.56 | 0.12 | 0.14 | 1.00 |  |
| Low income group | 0.09 | 0.03 | 0.01 | 0.09 | 0.16 | 0.02 | 0.05 | 0.15 | 1.00 |
